# Outcomes Associated with Lower Doses of Ketamine by Emergency Medical Services for Profound Agitation

**DOI:** 10.5811/westjem.2021.5.50845

**Published:** 2021-08-30

**Authors:** Shaila K. Coffey, J. Priyanka Vakkalanka, Haley Egan, Kelli Wallace, Karisa K. Harland, Nicholas M. Mohr, Azeemuddin Ahmed

**Affiliations:** * University of Iowa Carver College of Medicine, Department of Emergency Medicine, Iowa City, Iowa; † University of Iowa Carver College of Public Health, Department of Epidemiology, Iowa City, Iowa; ‡ University of Iowa Carver College of Medicine, Department of Anesthesia, Iowa City, Iowa; § University of Iowa Tippie College of Business, Iowa City, Iowa

## Abstract

**Introduction:**

Ketamine is commonly used to treat profound agitation in the prehospital setting. Early in ketamine’s prehospital use, intubation after arrival in the emergency department (ED) was frequent. We sought to measure the frequency of ED intubation at a Midwest academic medical center after prehospital ketamine use for profound agitation, hypothesizing that intubation has become less frequent as prehospital ketamine has become more common and prehospital dosing has improved.

**Methods:**

We conducted a retrospective cohort study of adult patients receiving ketamine in the prehospital setting for profound agitation and transported to a midwestern, 60,000-visit, Level 1 trauma center between January 1, 2017–March 1, 2021. We report descriptive analyses of patient-level prehospital clinical data and ED outcomes. The primary outcome was proportion of patients intubated in the ED.

**Results:**

A total of 78 patients received ketamine in the prehospital setting (69% male, mean age 36 years). Of the 42 (54%) admitted patients, 15 (36% of admissions) were admissions to the intensive care unit. Overall, 12% (95% confidence interval [CI]), 4.5–18.6%)] of patients were intubated, and indications included agitation (n = 4), airway protection not otherwise specified (n = 4), and respiratory failure (n = 1).

**Conclusion:**

Endotracheal intubation in the ED after prehospital ketamine use for profound agitation in our study sample was found to be less than previously reported.

## INTRODUCTION

Profound agitation is a high-risk medical condition that left untreated can progress to hypertension, tachycardia, hyperthermia, and altered mental status and can lead to rhabdomyolysis. The risk of death due to excited delirium syndrome has been reported to be between 8.3%–16.5%.^1^ Recognition and understanding of the disease have led emergency medical services (EMS) systems to develop commensurate treatment protocols.

Ketamine has emerged as a frontline medication in the treatment of profound agitation. Its intramuscular (IM) route of administration and short and predictable onset has led to widespread use in the EMS community.^2^

Burnett et al reported that complications such as hypoxia, laryngospasm, hypersalivation, and excessive depth of sedation were common after prehospital ketamine administration, and 15% of patients were intubated on emergency department (ED) arrival.^3^ Risk was further questioned by a cohort study reporting 63% of cases required intubation.^4^ A follow-up, prospective study identified 57% of cases intubated on ED arrival.^5^

The objective of this study was to measure the incidence of intubation after prehospital ketamine use. We hypothesized that intubation has become less frequent as ketamine has become more routinely used in the prehospital setting and prehospital dosing has improved.

## METHODS

### Study Design, Setting, and Sample

We conducted a retrospective cohort analysis of all adult patients (≥18 years) transferred by a single advanced life support (ALS) ambulance service with a catchment area of 623 square miles to a 60,000-visit midwestern university Level 1 trauma center between January 1, 2017–March 1, 2021. The service is the sole 911 ALS response agency in the catchment area transporting all qualifying patients to the study destination. We based the inclusion date on when EMS started using its current, discoverable charting system and ended when we reached our goal sample size according to our sample-size calculation. All patients receiving prehospital ketamine (ie, on scene or during transport) for profound agitation were included in the study. Local protocol allowed for the administration of 3 milligrams per kilogram (mg/kg) ketamine IM for adults exhibiting concerns of profound agitation.^6^ We obtained data from the ambulance medical record and the linked receiving hospital’s electronic health record (EHR) system. The local institutional review board approved this study under waiver of informed consent, and the study is reported in accordance with the Strengthening the Reporting of Observational Studies in Epidemiology (STROBE) guidelines.^7^

### Measurements of Exposures and Covariates of Interest

Data were extracted from medical records by two investigators using a standardized data collection form. Both investigators were unblinded (one medical student and one EMS physician familiar with both the local EMS and hospital EHR protocols). We entered data into an electronic database with standard data reporting formats (REDCap, Vanderbilt University, Nashville, TN).^8^ We selected prehospital charts based on a search criterion of ketamine medication administration. From that point, each chart was reviewed for indication of ketamine administration and was included in the study if ketamine had been administered for profound agitation defined as “patient exhibiting behavior (violent, combative, uncooperative) deemed to present a danger to self, EMS personnel, or bystanders despite verbal de-escalation attempts” per local prehospital protocols. Ketamine administration was abstracted from the ambulance medical record with the dose, route, need for redosing, and co-administration with other medications.

Demographic variables assessed included patient age, gender, and race. Selected comorbidities from the patients’ past medical histories included schizophrenia, depression, bipolar disorder, hypertension, asthma, and traumatic brain injury. The patients’ vital signs during their EMS transport as well as those measured in the ED were recorded. Blood pressure was categorized based on systolic blood pressure values (>160 millimeters mercury (mm Hg): hypertensive; 100–160 mm Hg: normotensive; and <100 mm Hg; hypotensive).

**Population Health Research Capsule**

What do we already know about this issue?

*Ketamine is commonly used to treat profound agitation in the prehospital setting. Early in ketamine’s prehospital use, intubation after arrival in the emergency department (ED) was frequent*.

What was the research question?

*What is the incidence of intubation after prehospital ketamine as it has become more routinely used in the prehospital setting?*

What was the major finding of the study?

*Intubation in the ED was found to be less than previously reported (12%), using 3.1 milligrams/kilogram prehospital ketamine dose*.

How does this improve population health?

*Ketamine has been associated with higher intubation rates rates previously. At lower doses it may still be an effective and safe option for prehospital sedation for profound agitation*.

We identified select medications administered in the ED that may have been associated with the outcome of interest such as benzodiazepines, opioids, or additional doses of ketamine. Weight-based dosing was based on EMS dose of ketamine divided by the actual measured weight obtained in the ED. Laboratory test results included blood glucose, lactate, venous blood gas, creatine kinase levels, and blood alcohol levels.

### Outcomes of Interest

The primary outcome was the proportion of patients intubated in the ED. If patients were intubated, we identified the primary indication for intubation (eg, agitation, airway protection, or respiratory failure) from the emergency physician note in the procedures section under “indication for procedure.” Secondary outcomes included presence of complications due to ketamine (eg, decreased level of consciousness, somnolence, and need for supplemental oxygen).

### Statistical Data Analysis

We reported patient demographics, comorbidities, and prehospital and ED vital signs descriptively. We estimated the proportion of patients who were intubated and a 95% binomial proportion confidence interval (CI) test. The means and standard deviations of ketamine dosing were calculated, and mean differences and 95% CIs are reported. We compared proportions of concomitant administration of benzodiazepines between intubated and non-intubated patients and reported relative risks and 95% CIs. Complications due to ketamine were assessed descriptively. For quality assurance, a 20% random sample of patients was generated for review of key study variables including intubation, EMS medications administered, ED medications administered, and ED disposition. A third study investigator independently assessed these charts and a kappa statistic (with 95% CI) was used to measure interrater agreement within this sample. Analyses were completed using SAS version 9.4 (SAS Institute, Cary, NC).

## RESULTS

A total of 95 patients received ketamine for profound agitation during the study period. Of those patients, two were excluded as they were transported to other receiving facilities, 14 were excluded because they were minors, and one was excluded as the patient did not have any patient-level identifiers. The final study sample included 78 patients who received ketamine in the prehospital setting for profound agitation during the study period (Figure). Demographics and clinical presentations are identified in Table 1, and vital statistics and laboratory values are presented in Table 2. Most patients were male (69%) and White (77%). Depression (32%) and other mental health diagnoses (28%) were prevalent in past medical history.

Overall, 12% (95% CI, 4.5, 18.6%) of patients were intubated, and indications for intubation included agitation (n = 4), airway protection not otherwised specified (NOS) (n = 4), and respiratory failure (n = 1). Possible complications for ketamine included the need for supplemental oxygen (n = 9), prolonged decreased level of consciousness (n = 1), and somnolence (n = 1). For patients whose weight had been recorded in the ED (n = 74), there was no difference in the average dose of ketamine between intubated (3.1 mg/kg) patients and non-intubated patients (3.0 mg/kg) (mean difference = 0.05; 95% CI, −0.68, 0.78). Of those patients who were intubated, 6 of 9 (67%) had received one or more doses of a benzodiazepine (not including benzodiazepines to assist with intubation) in addition to the ketamine either by EMS (n = 2), in the ED (n = 2), or by EMS and in the ED (n = 2). Among patients who were not intubated, 31 of 69 (45%) received additional benzodiazepines overall by EMS (n = 6), in the ED (n = 22), or by EMS and in the ED (n = 3). Overall, there was no significant difference in the odds of receiving concomitant administration of benzodiazepines between intubated and non-intubated patients (odds ratio [OR]: 1.48; 95% CI, 0.87, 2.52).

Thirty-six patients (46%) who received ketamine by prehospital personnel were discharged home directly from the ED, while 27 (35%) were admitted to the general medical floor and 15 (19%) required admission to the intensive care unit (ICU). Of the 15 patients admitted to the ICU, 6 (40%) were not intubated in the ED and did not require intubation in the ICU subsequently. The reason for ICU admission in the six non-intubated patients were concomitant foreign body ingestion (n = 1); ischemic stroke diagnosed by computed tomography (n = 1); significant anemia (n = 1); decreased mental status requiring close monitoring but not intubation (n = 1); psychosis with need for repeated intravenous sedation (n = 1); and hyperglycemia with concern for possible seizure (n = 1).

The results from the quality assurance review and interrater agreement are presented in Table 3. Briefly, nearly all components assessed had 100% concordance.

## DISCUSSION

In our study the proportion of patients intubated after receiving prehospital ketamine for profound agitation was lower than previously reported in the literature. Previous studies showed rates of intubation in the ED after prehospital ketamine administration at 23%–63%.^4^,^5^,^9^–^13^ Cole et al found that 57% of ketamine patients were intubated and over one-third of those intubations were attributed to one physician and that the night shift was a prognostic factor of intubation.^5^ They acknowledged that several studies that reported prehospital ketamine use for profound agitation were from their institution and may have been biased by their local practice variation.^4^,^9^ In studying our local practice, we found that the ED intubation proportion after administration of prehospital ketamine (12%) was much lower than previously reported and there was no specific association between certain providers or time of day and intubation proportion.

The lower intubation proportions are important because the prevalence of agitation in patients presenting to the ED has been quoted at 2.6%, with 84% requiring physical restraint and 72% requiring chemical sedation.^14^ Ketamine used for the treatment of profound agitation has a quick onset of action with peak sedation in less than five minutes.^15^ It is an effective sedating agent in the prehospital treatment of profound agitation with a 90% success rate.^5^ Its clinical effectiveness makes it suitable for use in the prehospital setting but must be weighed against its potential risks including intubation in the ED.

There may be several reasons why other reports noted larger proportions of patients receiving ED intubations after prehospital ketamine for profound agitation. Our local ambulance protocol suggested that EMS personnel administer 3 mg/kg doses of ketamine, which was on the lower end of the dosing scale compared to previous studies. In the published literature, the mean ketamine doses were between 4.9–5.3 mg/kg.^5^,^11^ The mean ketamine dose in our sample was 3.1 mg/kg. This lower dose as compared to previous studies may play a role in the decreased, all-cause ED intubation proportions after prehospital ketamine administration for profound agitation.

With a lower dose administered, there may be concern for decreased effectiveness. Upon further investigation of our patient sample, repeat dosing was needed in seven patients (9%) suggesting that the majority of patients were sedated adequately to allow safe transport with one dose (91%). One previous study used a similar mean ketamine dose (3.0 mg/kg) to ours, with a decreased intubation proportion of 8.7% while describing an adequate decrease in agitation with an average agitation score of 1.25 at five minutes.^16^ This study had a smaller sample size (n = 23), and its focus was not to estimate the proportion of intubated patients but to compare ketamine to other medications in the treatment of agitation in the ED. Another study with a lower dose of ketamine (3.8 mg/kg) also found similar results of decreased intubation proportion of 6.2%.^17^ These previous studies as well as ours, using a lower dose per kilogram of ketamine, suggest that a lower dose of ketamine may reduce intubation proportions.

Our reported proportion of patients who required redosing is similar to the reported proportion in the meta-analysis by Mankowitz et al.^18^ In their meta-analysis, they found that 24.4% of included patients required further sedation with either additional ketamine, benzodiazepine, or an antipsychotic. The mean ketamine dose administered throughout the included literature was 4.9 mg/kg, which was higher than our observed ketamine dose. This suggests that despite differing initial ketamine dosages, redosing and the need for additional sedation occurs and that higher initial dosage may not prevent the need for redosing.

Looking further at our data, we found no significant difference in the odds of receiving concomitant administration of benzodiazepines between intubated and non-intubated patients (OR: 1.48; 95% CI, 0.87, 2.52). Few other studies have explicitly addressed this; however, Olives et al did evaluate this concept in their study and reported similar results with no association between concomitant administration of further sedating medications in addition to ketamine and intubation proportions.^4^ In fact, some reports in the literature have suggested that benzodiazepines can be used to minimize emergence reactions.^19^ However, other studies have refuted this finding^20^ and have shown that benzodiazepines cause suppression of ketamine metabolism,^21^ which prolongs ketamine recovery time in addition to the dose-dependent respiratory depression that benzodiazepines cause.^22^ Our findings suggest that the addition of benzodiazepines to ketamine in the treatment of profound agitation does not increase the risk of intubation when compared to ketamine administration alone.

Cole et al found that the most common indication for intubation was “airway unprotected NOS,” which they identified as vague and suggested that there were other deciding factors, such as Glasgow Coma Scale (GCS), driving the decision to intubate.^5^ Ketamine produces a catatonic-like state in patients^23^ while having the unique properties of retained airway reflexes, hemodynamic stability, and maintenance of spontaneous respirations.^24^ Previously, emergency physicians may have observed a patient under the effects of ketamine with a GCS of <8 and intubated these patients solely due to decreased GCS when, in fact, a decreased GCS as a primary indication for intubation has been refuted.^25^

Another association that had been previously noted was the risk of intubation and time of day as well as the emergency physician performing the procedure. Cole et al found that one-third of their recorded intubations were attributed to one physician and that the night shift was a prognostic factor of intubation.^5^ In our sample size, we did not see similar results. A later time of day (9 pm – 7 am) occurred in four out of nine intubations, an additional four intubations occurred between 7 am – 3 pm, and one intubation occurred between 3 pm – 9 pm. With regard to physicians at our facility, only two of the nine intubations were performed by one physician and the indication for intubation in both was agitation.

Prehospital use of ketamine for profound agitation has previously been associated with heterogenous results such as hypoxia, hypersalivation, and high intubation rates^3^,^5^; however, our study has shown that few prehospital ketamine patients require endotracheal intubation. This finding suggests that prehospital personnel can more comfortably consider the use of ketamine in the treatment of profound agitation while they customize their care to individual patients, give lower doses of ketamine, and avoid concomitant doses of benzodiazepines.

## LIMITATIONS

There are several limitations to consider. First, as a retrospective study there may be unmeasured confounding variables. Details of the prehospital presentation were often incomplete, and we could only measure associations and not causation. Being limited to only what was documented in the EHR, we were unable to obtain in-depth description of the patients’ mental status longitudinally during their ED stay. The low frequency of profound agitation occurrence and limited availability of cases restricted our sample size and thereby limit our ability to make broad, generalizable conclusions. In addition, as a single-center study, our results may only infer local practice variations.

Another limitation is that we were unable to directly discuss the indication for intubation and the details of decision-making behind intubation after prehospital ketamine administration for profound agitation, as was done in a few other studies. Due to the retrospective design of our study, we were unable to have such conversations with the emergency physicians performing the intubations to have this insight. This led to less knowledge of the circumstances driving the decision to intubate. Lastly, chart review was performed by two people, and only a 20% sample was reviewed for interrater agreement. Because our criteria for inclusion and outcomes were objective (ketamine use vs not, intubation vs not, disposition type, medications administered) this objectivity decreases this risk but does not eliminate it. Review of the interrater agreement with high concordance across several measures alleviated concerns associated with manual data extraction.

## CONCLUSION

The incidence of intubation after prehospital ketamine in this single-center, retrospective review was found to be less frequent then previously reported. This result may be because ketamine has become more routinely used in the prehospital setting with decreased prehospital dosing.

## Figures and Tables

**Figure:**
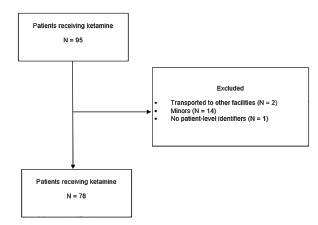
Flow chart of study sample.

**Table 1:** Characteristics of patients receiving ketamine in the prehospital setting.

Demographics and clinical history		
Age - mean (SD)	36	(15.3)
Male - N (%)	54	(69.2)
Race - N (%)		
White/Caucasian	60	(76.9)
Black/African American	10	(12.8)
Asian	6	(7.7)
Other	2	(2.6)
Previous medical history - N (%)		
Bipolar disorder	12	(15.4)
Schizophrenia	8	(10.3)
Depression	25	(32.1)
Other mental health diagnosis	22	(28.2)
Hypertension	11	(14.1)
Asthma	8	(10.3)
Traumatic brain injury	3	(3.9)
Clinical management and outcomes		
EMS medication administration - N (%)		
Benzodiazepines	13	(16.7)
Other	1	(1.3)
ED medication administration - N (%)		
Benzodiazepines	29	(37.2)
Ketamine	7	(9.0)
Other	17	(21.8)
Intubation - N (%)	9	(11.5)
Urine drug screen results^1^		
Amphetamines	21	(41.2)
Opioids	1	(2.0)
Other	11	(21.6)
THC	20	(39.2)
Hospital disposition - N (%)		
ICU admission	15	(19.2)
Inpatient admission	27	(34.6)
Discharge	36	(46.2)

1 Among 51 patients who had a urine drug screening performed.

*ED*, emergency department; *EMS*, emergency medical services; *THC*, tetrahydrocannabinol; *ICU*, intensive care unit.

**Table 2:** Vital statistics and laboratory test values for patients receiving ketamine in the prehospital setting.

Measure	N included^1^	Mean	SD
EMS Vitals			
Temperature (°F)	18	99.1	(1.7)
Heart rate (beats per minute)	74	121	(23.2)
Respiratory rate (breaths per minute)	67	21	(6.5)
Pulse oximetry (%)	66	95.1	(8.5)
Blood pressure^2^			
Hypertensive		29	(37.2)
Normotensive	78	32	(41.0)
Hypotensive		1	(1.3)
Missing		16	(20.5)
ED vitals			
Temperature (°F)	75	98.6	(1.2)
Heart rate (beats per minute)	77	108.7	(23.7)
Respiratory rate (breaths per minute)	76	20.3	(8.9)
Pulse oximetry (%)	77	95.7	(3.3)
Blood pressure			
Hypertensive		21	(26.9)
Normotensive	78	57	(73.1)
Hypotensive		0	(0.0)
Ketamine dosage administered (mg/kg)	74	3.1	(1.1)
Blood glucose (mg/dL)	74	142.2	(107.1)
Lactate (mmol/L)	25	4.7	(4.8)
Venous blood gas			
pH	22	7.3	(0.1)
pCO_2_	22	44.7	(8.1)
pO_2_	22	99.3	(80.6)
Bicarbonate (mmol/L)	22	20.7	(4.5)
Creatine kinase (U/L)	22	849.2	(943.5)
Blood alcohol level (mg/dL)	66	80.3	(119.8)

1 Refers to number of patients with a value reported.

2 Blood pressure was categorized based on systolic blood pressure values (>160 – hypertensive, 100–160 – normotensive, and <100 – hypotensive).

*SD*, standard deviation; *ED*, emergency department; *EMS*, emergency medical services; *F*, Fahrenheit; *mg/kg*, milligrams per kilogram; *THC*, tetrahydrocannabinol; *pCO_2_*, partial pressure of carbon dioxide; *pO_2_*, partial pressure of oxygen; *mmol/L*, millimoles per liter; *U/L*, units per liter; *mg/dL*, milligrams per deciliter.

**Table 3:** Assessment of 20% sample for interrater agreement (N = 16).

Measure	Kappa statistic^1^	95% CI
Intubation	1.0	1.0–1.0
EMS medications		
Benzodiazepines	1.0	1.0–1.0
Other	1.0	1.0–1.0
ED medications		
Benzodiazepines	1.0	1.0–1.0
More ketamine	1.0	1.0–1.0
Other	0.7	0.3–1.0
ED disposition		
ICU admission	1.0	1.0–1.0
Inpatient admission	1.0	1.0–1.0
Discharge	1.0	1.0–1.0

1 Kappa statistic of 1 presented indicate no discordant pairs between the two data abstractors.

*CI*, confidence interval; *ED*, emergency department; *EMS*, emergency medical services; ICU, intensive care unit.
